# Effect of deep diaphragmatic breathing on pain in patients with metastatic gastrointestinal cancers

**DOI:** 10.1016/j.heliyon.2024.e40283

**Published:** 2024-11-09

**Authors:** Maryam Rezaei, Nader Salari, Mozafar Aznab, Sayed Vahid Jasmi, Alireza Abdi, Shamarina Shohaimi

**Affiliations:** a Department of Emergency and Critical Care Nursing, School of Nursing and Midwifery, Student Research Committee, Kermanshah University of Medical Sciences, Kermanshah, Iran; b Department of Biostatistics, School of Health, Kermanshah University of Medical Sciences, Kermanshah, Iran; c Department of Internal Medicine, School of Medicine, Taleghani Hospital, Imam Reza Hospital Kermanshah University of Medical Sciences, Kermanshah, Iran; d Department of Internal Medicine, School of Medicine, Imam Reza Hospital, Kermanshah University of Medical Sciences, Kermanshah, Iran; e Department of Emergency and Critical Care Nursing, Nursing and Midwifery School, Kermanshah University of Medical Sciences, Kermanshah, Iran; f Department of Biology, University Putra Malaysia, Serdang, Selangor, Malaysia

**Keywords:** Gastrointestinal cancers, Deep diaphragmatic breathing, Pain, Nursing

## Abstract

**Background:**

Patients with gastrointestinal cancers commonly experience acute and chronic pain. This study aimed to determine the effect of deep diaphragmatic breathing on acute and chronic pain in patients with metastatic gastrointestinal cancers.

**Methods:**

This clinical trial was conducted in Kermanshah-Iran in 2022. The sample consisted of 44 patients hospitalized in the oncology ward, who were selected by convenient sampling and randomly allocated into case and control groups. The case group performed diaphragmatic deep breathing intervention for 10 days, twice a day, for 10 min. The subjects completed pain assessment tools before and after the intervention. Data were analyzed using SPSS Version 24.

**Results:**

The study included participants with a Mean and Standard Deviation(SD) age of 53.95 ± 10.51 years. The case and control groups were similar in terms of demographic variables. The mean and sd acute pain score before the intervention was 3.50 ± 1.84 in the experimental group and 2.18 ± 1.65 in the control group (p = 0.01). However, after the intervention, the score decreased to 1.72 ± 1.07 in the experimental group and increased to 3.72 ± 1.95 in the control group (p = 0.001). The two groups did not differ significantly in terms of chronic pain before the intervention (p = 0.07). However, after the intervention, the score decreased in the experimental group and increased in the control group, with a significant difference (p = 0.01).

**Conclusion:**

The results of this study suggest that deep diaphragmatic breathing reduces pain in patients with gastrointestinal cancer. Including this method as a routine care program for cancer patients is recommended.

## 1 Background

Cancer is a group of diseases characterized by the uncontrolled growth of cells in the body. It is caused by a combination of genetic and environmental factors and is a leading cause of death and disability worldwide [1]. According to the latest report of the World Health Organization, nearly 7 million people die from cancer each year globally [2]. Among the various types of cancer, gastrointestinal (GI) cancers, including colon and stomach cancer, have high mortality rates [3]. Cancer patients often experience distressing symptoms [4], and pain is a common problem among them [5].

Pain is an unpleasant sensory experience associated with actual or potential tissue damage. It can be classified as acute or chronic based on its duration. Acute pain lasts for less than three months, while chronic pain typically persists for more than three to six months [6]. Chronic or long-term pain can cause changes in the levels of neurotransmitters, neuromodulating lipids, and neuropeptides, which in turn creates neural plasticity. These changes can not only affect the structure of the brain but can also have an impact on cognitive functions such as learning, memory, problem-solving, and processing speed. Moreover, they can lead to mood and emotional disorders, including depression [7]. The biopsychosocial model recognizes the biological, psychological, and social components of pain and illness. It accounts for the dynamic interactions among these factors and emphasizes how individuals live with and respond to symptoms. However, the model requires further adaptation to provide more effective pain management approaches. Interdisciplinary assessment and treatment of chronic conditions are necessary, and unique symptom patterns necessitate tailored interdisciplinary pain management programs for each patient [8].

Cancer pain is a complex phenomenon involving varying levels of acute and chronic pain caused by the aggressive spread of tumor cells throughout the body [9]. Acute pain can activate the sympathetic branch of the central nervous system, leading to physiological reactions such as increased blood pressure, tachycardia, shallow breathing, and psychological symptoms [6]. Acute pain can be caused by tumor bleeding, microsites, polyneuropathy, headaches, cardiac toxicity, deep vein thrombosis, abdominal pain, phlebitis, and chemotherapy drug extravasation [10]. While acute pain is usually relieved within a few days to weeks, inadequate management of acute pain can contribute to the development of chronic pain. Chronic pain does not usually respond to conventional treatments [6]. The most common causes of chronic pain in cancer patients include bone and liver metastases, chronic intestinal obstruction, perineal pain, and malignant ureteral obstruction [10].

Against the previous beliefs, the brain is a highly dynamic organ, constantly adapting to new conditions through a process called neuroplasticity. One factor contributing to chronic pain is structural changes in the brain. As chronic pain persists, it can impact both the volume and activity of the brain, leading to changes in the relationship between various regions and pain pathways. Over time, pain representation may shift from sensory structures to areas responsible for emotional responses and the limbic system. This can result in more complex emotional and cognitive states, as reflected by increased activation in the frontal lobe [7]. Chronic and acute pain can lead to the continuous production of corticosteroid hormones such as cortisol, suppressing the immune system and increasing the risk of infection [11].

The American Pain Society has recommended assessing and diagnosing pain as the fifth vital sign [12]. However, almost 40–50 % of cancer pain cases do not receive adequate relief due to their multifactorial nature [9]. Pain management usually involves the use of painkillers [10]. Medication options include narcotics for physical pain and anticonvulsants for nerve pain. These drugs may have side effects such as drowsiness, constipation, and nausea. Acetaminophen and nonsteroidal anti-inflammatory drugs are used for mild to moderate pain but are not recommended during chemotherapy as they can mask the symptoms of fever, which serve as warning signs of infection. The Joint Commission of the American College of Physicians (ACP), the National Comprehensive Cancer Network (NCCN), and the American Society of Clinical Oncology (ASCO) all recommend a combination of pharmacological and non-pharmacological methods to manage the challenges faced by cancer patients [13].

Relaxation methods, including relaxation/breathing exercises, are an integral part of supportive and non-pharmacological care and have been used in various types of cancer and have significant benefits [14]. Breathing techniques can be categorized as thoracic or abdominal breathing, with deep diaphragmatic breathing being one approach [15]. The relationship between the diaphragm and chest movement during breathing was first investigated by Osval and Pollard in the late 19th Century [16]. The diaphragm muscle, separating the chest from the abdominal cavity, is crucial for breathing [17]. Deep diaphragmatic breathing is one of the oldest relaxation techniques that reduces the activity of the sympathetic nervous system and increases the activity of the parasympathetic nervous system [18], thereby exerting a significant effect on chronic pain [19]. This method is advantageous due to its safety, simplicity, and the ability to be implemented anytime and anywhere [18]. Limited studies have examined the effect of deep and calm breathing on pain in cancer patients, and the findings from studies involving other patient populations are conflicting. For instance, a study by Larsen et al., in 2019 [20] demonstrated that deep, slow breathing alone is insufficient for pain reduction.

Similarly, the study by Gholamrezaei et al., in 2021 [21] also showed that slow and deep breathing does not significantly affect pain reduction. Additionally, the study by Downey et al., in 2009 [22] indicated that deep breathing does not impact pain reduction. Given the need for pain relief in patients with GI cancer as part of palliative care, and considering the limited research in this area, the present study aims to determine the effect of deep diaphragmatic breathing on acute and chronic pain in patients with metastatic GI cancers.

## 2 Methods

**Fig. 1:**
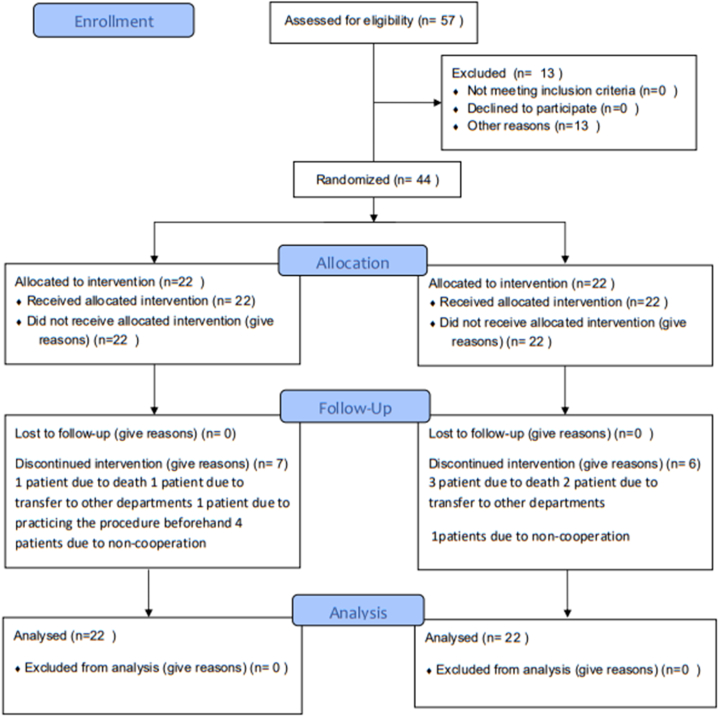
Consort flowchart diagram.

This clinical trial was conducted at the oncology departments of Imam Reza (AS) Kermanshah Hospital, the largest medical center in the west of Iran, from 2021 to 2022. The study followed the CONSORT guidelines of 2010 (Fig. 1). The research population consisted of all patients admitted to the oncology departments of Imam Reza Hospital-Kermanshah. The sample included 44 patients with metastatic gastrointestinal (GI) cancers. The inclusion criteria were a diagnosis of metastatic cancer of the GI based on the doctor's diagnosis, the patient's willingness to participate, age between 18 and 65 years, and the experience of periodic pain. Exclusion criteria included transfer from the oncology department to other units, death, misdiagnosis, drug abuse, and patients with decreased consciousness levels. The patients did not get any other non-pharmacological measure for pain relief, and their painkillers were based on the ward routines including pethidine, morphine, and paracetamol.

The patients were conveniently selected and randomly assigned to two groups: the case group (deep diaphragmatic breathing) and the control group (receivers of normal care). Random assignment was performed using identical cards, with 22 cards labeled with the letter A for the case group (indicating “deep diaphragmatic breathing") and 22 cards labeled with the letter B for the control group (indicating "usual care"). Another person would randomly choose one of these cards on which the codes were written to determine the random assignment of patients to each group.

The sample size of 17 individuals per group was determined based on the pain variable findings reported in studies by Gopichandran et al. (2021) [23] and Lalgani et al. (2013) [12]. This calculation utilized a formula for comparing quantitative traits between two groups, with a confidence coefficient of 95 % (α-1) and a test power of 90 % (β-1).

To obtain more accurate results and account for possible attrition, a minimum of 22 individuals per group was determined, resulting in a total of 44 patients for this study. Data collection involved the researcher obtaining permission from the Vice-Chancellor of Research and Information Technology at Kermanshah University of Medical Sciences and the hospital officials. The researcher then visited the oncology departments and selected individuals who met the inclusion criteria. After comprehensively explaining the research method and objectives, the patients were asked to sign the informed consent form. Data collection lasted approximately three months, from March 2021 to May 2022. The information was gathered using a designed checklist through patient records and interviews.

### 2.1 Data collection tools

This study utilized the following tools: 1: Patient demographic information checklist. This checklist captured relevant factors in cancer patients, including age, gender, occupation, health insurance status, medical history (including cancer type and metastasis location), treatment regimen, and family history of cancer. 2: A visual analog scale was utilized to assess acute pain, employing a 10-cm line to depict a spectrum from 'no pain (0)' to 'most severe pain [10]. Participants in the study were asked to indicate the point on the scale or provide the corresponding number that represented their pain intensity. This widely-used tool has established validity and reliability, including in various studies conducted in Iran, such as the study on the effect of diaphragmatic breathing on pain in burn patients [12]. 3: "The VanKorff Chronic Pain Questionnaire, established by VanKorff et al., in 1992, is utilized to evaluate chronic pain [24]. It encompasses the assessment of chronic pain intensity, duration or stability of pain (days of disability), and the level or degree of disability resulting from pain.

Levels or degrees of disability are classified based on pain intensity and disability score as follows.

Level 0 = zero pain intensity and zero disability score
Level 1 = pain intensity< 50 and disability score > 3 (low disability - low intensity)
Level 2 = pain intensity ≥50 and disability score > 3 (low disability - high intensity)
Level 3 = disability score of 3 or 4 regardless of pain intensity (high disability - moderate limitation)
Level 4 = disability scores of 5 or 6 regardless of pain intensity (high disability - severe limitation)

Based on this scale, there is a direct relationship between the grade obtained and the degree of disability.

Participants rate each of the seven question statements on an eleven-point scale ranging from 0 to 10. The test provides scores in three subscales: pain intensity, duration or stability of pain(days of disability), and Levels or degrees of disability. Total chronic pain is calculated from the sum of three subscales and interpreted as follows:

A total score between 0 and 20: low chronic pain.

A total score between 20 and 35: Moderate chronic pain.

A total score above 35: high chronic pain [24]. The validity and reliability of this questionnaire have also been demonstrated in an Iranian study titled "Effectiveness of group psychotherapy based on acceptance and commitment to reducing pain intensity in patients with chronic pain" [25].

### 2.2 Intervention

The experimental group underwent a diaphragmatic breathing intervention in this study based on various sources. These sources include Hayama's study titled “The Effect of Deep Breathing on Tension, Anxiety, and Fatigue in Patients Undergoing Adjunct Chemotherapy [26], Hamsky's study on the effect of diaphragmatic breathing on health(2020) [27], and a citation from the London Pain Clinic website [28]. The diaphragmatic breathing intervention was performed twice a day for ten consecutive days.

At the end of the 19th century, Osval and Pollard first investigated the relationship between the movement of the diaphragm and the chest during breathing [16]. The diaphragm muscle separates the chest from the abdominal cavity and is the most important muscle in breathing. In addition to ventilation, the diaphragm has other important functions. It is necessary for better heart function, venous and lymphatic return, gastroesophageal function (vomiting, swallowing), regulation of emotional states, and pain threshold [17]. The deep diaphragmatic breathing technique was performed in both the lying and sitting positions using the following steps.

Step 1: Subjects were instructed to lie comfortably on the bed, with the option to bend their knees and use a pillow under their legs if needed. Alternatively, they could sit comfortably. One hand was placed on the upper chest, and the other on the abdomen.
Step 2: Subjects were instructed to keep their tongue motionless at the bottom of the mouth.
Step 3: Subjects were educated on taking a deep breath for 2–5 s.
Step 4: They were encouraged to hold their breath for 5-2 s.
Step 5: Patients were instructed to exhale slowly through the nose for 2–5 s.

To enhance relaxation and prevent distraction, providing a calm environment and avoiding speaking during the process [13,21,29].

### 2.3 Data collection from the intervention group

In the case group, patients were provided with instruction on how to perform slow and deep diaphragmatic breathing until they achieved complete mastery. This was done through the use of pamphlets, educational videos, and face-to-face training. Before the intervention, the research instruments were completed by the patients. The patients then performed breathing exercises for 10 min twice a day (at 9 a.m. and 9 p.m.) for 10 days. Ten days after the intervention, the patients again completed the questionnaires for comparison.

### 2.4 Collect data from the control group

In the control group, no training was provided on performing deep diaphragmatic breathing. The patients in this group received regular care from the ward. Similar to the intervention group, the desired variables were measured using the research tools before the start of the intervention. After 10 days, the questionnaires were completed by the individuals in the control group for comparison.

### 2.5 Ethical considerations

This study has been registered in Iran's clinical trial system with the clinical trial code (IRCT20220104053628N1). Approval was obtained from the Ethics Committee of Kermanshah University of Medical Sciences with license number (kums.rec.1400.759), and written informed consent was obtained from all the patients. Assurance regarding the confidentiality of patient information was provided to both the patients and the hospital officials. At the end of the study, the deep diaphragmatic breathing program was provided to the control group.

### 2.6 Data analysis

Statistical analysis was performed using SPSS Version 24 software. Descriptive statistics were used to analyze the demographic information of the two groups based on qualitative variables (such as gender, marital status, education level, and occupation) using the chi-square test. The normality of quantitative variables was assessed using Shapiro-Wilk's normality test. Based on this test, quantitative demographic variables, as well as variables related to acute pain, chronic pain intensity, and total chronic pain, were found to be normally distributed. Therefore, parametric tests were used for analysis. However, variables related to the level or degree of disability and days of disability resulting from chronic pain were found to be non-normal, so non-parametric tests were used. Wilcoxon and paired t-tests were used to compare the mean rank and mean of quantitative variables before and after the intervention in each control and test group. The independent Mann-Whitney U and t-tests were used to compare the mean rank and mean of quantitative variables between the control and experimental groups before and in the post-intervention phase. The significance level for all tests was set at less than 0.05.

## 3 Results

In this study, a total of 57 patients were initially included. However, 13 patients (22 %) were subsequently excluded from the study. Among the exclusions, four patients (one from the experimental group and 3 from the control group) passed away during the course of the study, and three patients (one from the experimental group and two from the control group) were transferred to the COVID-19 and ICU departments. Additionally, one patient from the intervention group could not continue cooperation due to prior practice of the procedure, and five patients (4 from the intervention group and one from the control group) could not continue cooperation due to reasons such as depression and disappointment with the treatment. Therefore, the analysis was performed on 44 patients (78 % of the initial sample). Among the participants, 72.2 % were male, and 27.3 % were female. The average age of the samples was 53.95 ± 10.51 years, this measure was 51.63 ± 11.95 and 56.27 ± 8.49 years for case and control groups, respectively with no meaningful difference (t = 1.48 P = 0.146). In terms of cancer type, 38.6 % had intestine cancer. The lung was the most common site of metastasis for both groups at 22.7 %. Most individuals in both groups, accounting for 72.2 %, were married. In terms of employment status, the highest proportion of participants (31.8 %) were unemployed in both groups. Based on the chi-square test, the participants were generally homogeneous regarding demographic variables (Table 1).

**Table 1:** Demographic characteristics of the two groups based on gender, marital status and occupation.

Variables			group			
			Case	Control	Total	Statistical test
			N (%)	N (%)	N (%)	
1	Sex	Female	5(22.7)	7(31.8)	12(27.3)	$x^{2}=0.45$
		Male	17(77.3)	15(68.2)	32(72.7)	P = 0.73
2	Marital status	Single	5(22.7)	1(4.5)	6(13.6)	*X*^*2*^*=6.66* P = 0.08
		Married	16(72.7)	16(72.7)	32(72.7)	
		Divorced	0(0)	4(18.2)	4(9.1)	
		Widow	1(4.5)	1(4.5)	2(4.5)	
3	Employment status	Unemployed	7(31.8)	7(31.8)	14(31.8)	$x^{2}=1.4$ P = 0.7
		Housewife	4(18.2)	7(31.8)	11(25)	
		Employee	5(22.7)	3(13.6)	8(18.2)	
		Free job	6(27.3)	5(22.7)	11(25)	
4	Cancer type	Intestine	9 (40.9)	8 (36.4)	17 (38.6)	Fisher exact's test = 9.20 P = 0.153
		liver	1 (4.5)	1 (4.5)	2 (4.5)	
		Stomach	6 (27.3)	7 (31.8)	13 (29.5)	
		Gallbladder	0 (0.0)	1 (4.5)	1 (2.3)	
		Pancrease	1 (4.5)	2 (9.1)	3 (6.8)	
		Esophagus	0 (0.0)	2 (9.1)	3 (6.8)	
		Colon	5 (0.0)	0 (0.0)	5 (11.4)	
		adenocarcinoma	0 (0.0)	1 (4.5)	1 (2.3)	

In this study, the average acute pain before the intervention in the intervention group was 3.50 ± 1.84, which was higher than the control group's average of 2.18 ± 1.65 (p-value = 0.01). However, at the end of the study, the acute pain decreased to 1.72 ± 1.07in the intervention group and increased to 3.72 ± 1.95 in the control group. This difference was significant based on the independent *t*-test (p = 0.001) (Table 2). Regarding total chronic pain, the mean and SD before the intervention was 33.40 ± 11.73 in the experimental group, which was higher than the control's group mean of 27.31 ± 10.46(p = 0.07). However, after the intervention, the total chronic pain decreased to 25.18 ± 12.97 in the experimental group and increased to 34.40 ± 11.99in the control group. The difference after the intervention was significant in both groups according to the independent *t*-test (p = 0.01) (Table 3).

**Table 2:** Comparison of the overall mean of acute pain before and after the intervention in two groups.

Variables		group		
		Average	standard deviation	Statistical test
Acute pain before intervention	Case	3.5	1.84	t = 2.49
	control	2.18	1.65	ap = 0.01
Acute pain after inthe tervention	Case	1.72	1.07	t = -4.2
	control	3.72	1.95	ap = 0.001

a Is significant.

**Table 3:** Comparison of the overall mean of Chronic pain before and after the intervention in two groups.

Variables		group	standard deviation	Statistical test
		Average		
Chronic pain before intervention	Case	33.4	11.73	t = 1.81
	control	27.31	10.46	p = 0.07
Chronic pain after the intervention	Case	25.18	12.97	t = -2.44
	control	34.4	11.99	ap = 0.01

a is significant.

Three aspects of chronic pain were measured: intensity of chronic pain, duration or stability(days of disability) of pain, and the level or degree of disability resulting from pain. In the intervention group, there was a significant decrease in pain intensity from 47.42 ± 13.60 to 36.02 ± 16.10 (p = 0.001), while in the control group, the average increased from 34.84 ± 19.93±20.22 to 46.81 (p = 0.001).

Regarding the pain duration subscale (days of disability), the average in the test group was 0.95 ± 1.25 before the intervention, which decreased to 0.68 ± 1.12 after the intervention (P = 0.03). While the average change in the control group from 0.40 ± 0.85 to 0.63 ± 0.90 was not significant (p = 0.09).

Regarding the level or degree of disability, the intervention group reduced the average from 2.04 ± 1.04 before to 1.5 ± 1.18 after the intervention (p = 0.03). In contrast, in the control group, this average increased from 1.59 ± 1.09 to 2.04 ± 0.99(p = 0.02).

**Fig. 2:**
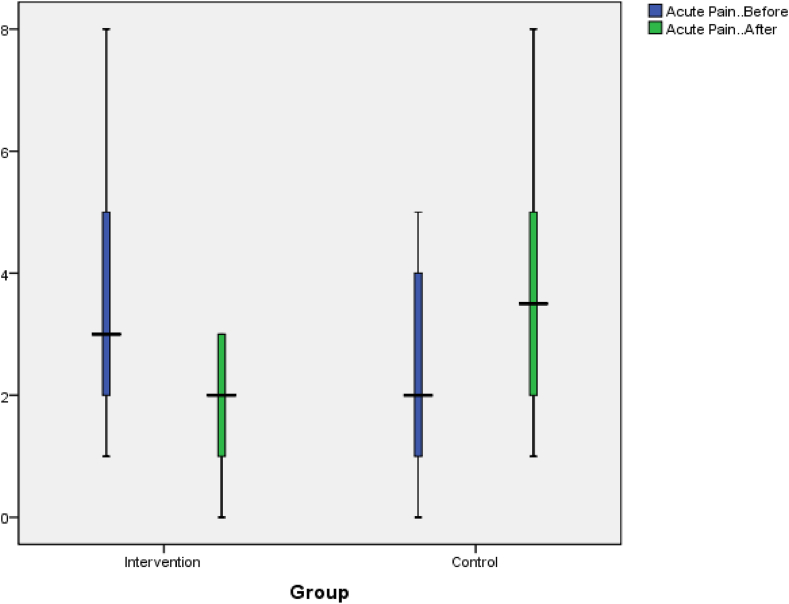
Acute pain changes in the test and control groups before and after the intervention.

**Fig. 3:**
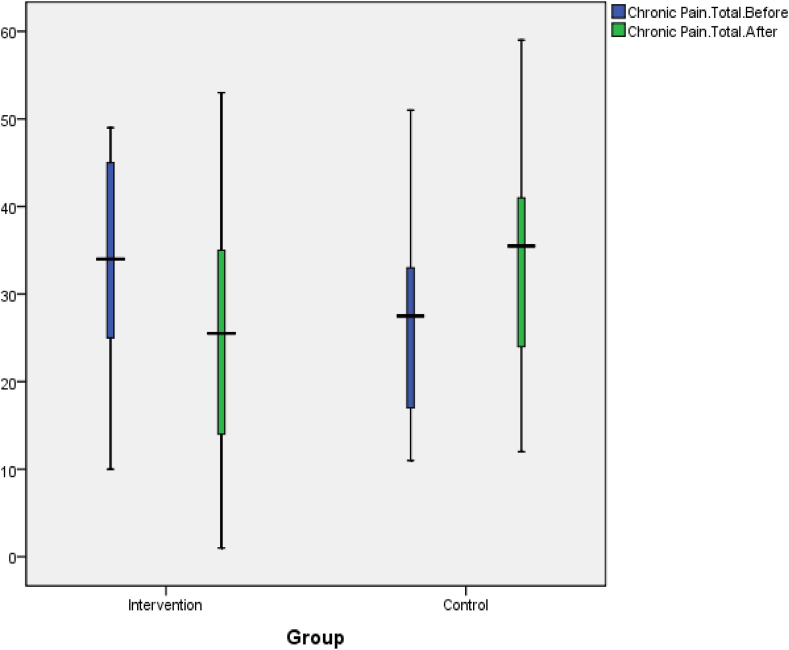
Changes in total chronic pain in the test and control groups before and after the intervention.

The changes in acute and chronic pain in both groups are illustrated in Fig. 2, Fig. 3.

## 4 Discussion

In this study, deep diaphragmatic breathing after ten days reduced acute pain, while in the control group, this variable has been increased significantly. Various studies indicate that the exact physiological and neurobiological mechanisms responsible for pain reduction through breathing have not been fully determined. However, it is believed that the cardiovascular system, particularly the baroreceptor and central pain processing systems, may play an important role. The baroreceptor system consists of a cardiovascular and main branch. Baroreceptors located in carotid sinuses, aortic arches, and heart cavities detect changes in blood pressure and heart rate during the respiratory cycle [[30], [31], [32]]. The vagus and glossopharyngeal nerves transmit these changes to the nucleus of the solitary duct (NST) located in the dorsal basal ganglia. NST fibers then connect with other parasympathetic and sympathetic brainstem nuclei, influencing sympathetic and parasympathetic cardiovascular tones, respectively [33]. Deep breathing may relieve pain by transferring changes in cardiovascular activity to brain areas responsible for regulating autonomic tone, processing pain stimuli, and modulating pain emotions [34]. These findings are consistent with a systematic review conducted by Wong et al. in Australia in 2022, which examined ten clinical trial studies and concluded that deep diaphragmatic breathing effectively alleviates pain in cancer patients [29]. Another study by Ozhanli et al., conducted in Istanbul, Turkey, in 2019 on 63 patients with colorectal cancer [34], also demonstrated a decrease in pain in the intervention group and an increase in the control group after the intervention. However, a study conducted in Sydney in 2021 investigating the relationship between breathing and pain [21] showed that slow and deep breathing positively impacted heart rate but did not significantly affect pain reduction in patients undergoing intervention. The discrepancy in the study outcomes may be due to the focus solely on the inspiratory part of breathing. In contrast, most similar studies emphasize the importance of the entire respiratory cycle [5,12,27]. For instance, a study conducted by Ragheb et al. in Egypt in 2014 on 70 children with cancer undergoing chemotherapy [35] found that children who had not undergone breathing exercises experienced more pain after the intervention than those who received breathing exercises. These results align with the findings of our study. The absence of pain control can lead to various physiological and psychosocial consequences for patients. Acute unresolved pain can result in depression, decreased quality of life, prolonged stress response, and difficulty accepting the disease and treatment measures. Pain also increases the body's metabolism, which can lead to malnutrition and immune deficiency [36].

Our results indicated that deep diaphragmatic breathing declined chronic pain during ten days, while in the control group, it increased significantly. According to previous research, relaxation methods have been found to enhance immune system activity, increasing the number of active T cells and the activity of NK cells, reducing inflammation, and providing pain relief [9]. The results of a 2020 He et al. study in China, which was a systematic review and meta-analysis study examining the effect of therapeutic breathing on non-specific low back pain [37], also indicated that patients who were deprived of the benefits of the respiratory method experienced higher rates of low back pain compared to patients undergoing intervention. The results of these studies are in line with the present study. Similarly, the results of Gopichandran et al.'s study in 2021 in India that examined the effectiveness of progressive muscle relaxation and deep breathing exercises on pain, disability, and sleep in patients with tension headaches [23], showed that the control group patients who did not perform muscle relaxation and deep breathing exercises had higher pain intensity and poor sleep quality after 12 weeks. However, the results of the Larsen et al. study in 2019 conducted in the U.S. titled “The Effect of Deep Slow Breathing on Pain-Related Variables in Osteoarthritis” [20] showed that the six-week deep, slow breathing program was insufficient to reduce pain or improve physical function in individuals with lower extremity joint pain, which contradicts the findings of our study.

To better understand chronic pain, we mention several theories. Melzack's neuromatrix theory posits pain as a multifaceted experience comprising sensory, emotional, and cognitive dimensions. Sensory signals, carried by nerve fibers, transmit physical pain sensations to the brain. The emotional aspect encompasses pain's impact on mood and well-being, while the cognitive facet involves interpretation, meaning-making, and coping mechanisms. Consequently, pain perception isn't solely determined by tissue damage but also by internal brain factors. Disorders affecting the neuromatrix may contribute to chronic pain conditions [38]. Melzack and Wall's Gate Control Theory encompasses physiological, psychological, cognitive, and pain sensation aspects, explaining how thoughts, emotions, and behavioral practices influence pain perception [7]. Piran's developmental theory of embodiment provides insight into the link between body image and chronic pain. It suggests that chronic pain experiences shape individuals' embodied self-perceptions. Consequently, chronic pain may affect body image by eliciting discomfort, self-consciousness, and dissatisfaction with one's appearance. Additionally, body image isn't solely influenced by external factors like societal norms but also by internal experiences, such as pain [39]. Since pain is not solely a sensory experience, it is important to consider non-pharmacological methods such as slow and deep breathing and medications to alleviate patients' discomfort and pain [12].

In this study, the two groups had no significant differences regarding demographic variables and disease-related factors before the intervention. Based on the results, it can be inferred that the increase in pain in the control group after the intervention is their lack of access to the benefits of deep diaphragmatic breathing. Consequently, the control group, who did not undergo the deep diaphragmatic breathing intervention during the study period, experienced various pain-related complications and challenges compared to the experimental group, which received the intervention, resulting in better pain control.

### 4.1 Limitations

There were certain limitations in conducting this research. The researcher could not observe the implementation of deep diaphragmatic breathing because the patients would return home after chemotherapy, and to remove this limitation, the patient and one of the family members would be trained until complete learning. Also, the implementation of the intervention at home was evaluated using a checklist and a telephone interview. The researcher initially had a limited understanding of the correct technique of deep diaphragmatic breathing. To address this limitation, an extensive review of relevant articles and books) for example The Book of Elixir of Youth and Longevity, written by Dr. Nobowashivia, as well as related articles such as the study of Martarelli et al. [14], the study of Kocjan et al. [16], the study of Sajjadi et al. [21] was used undertaken to acquire the necessary knowledge. During the study, there were instances of sample dropout, which prompted measures to address this limitation by increasing the sample size. Initially, there were challenges in obtaining full cooperation from the patients due to the nature of the disease and the presence of mental disorders such as depression. To mitigate this issue, additional time was allocated to explain the intervention's objectives, methods, and benefits to the patients. Moreover, oncologists initially had reservations regarding the efficacy of complementary medicine in addressing patient issues. To solve this problem, several articles and books concerning the intervention were presented to the oncologists, which led to their willingness to cooperate and agree to implement the intervention in the patients under treatment. It should be noted that in both groups, pain relief was done outside of the study, using routine measures, including the use of a Pain reliever.

## 5 Conclusion

The purpose of this study was to investigate the effect of diaphragmatic breathing on acute and chronic pain in patients with metastatic GI cancers. The results of this study demonstrated that the deep diaphragmatic breathing method, performed twice a day for 10 min throughout 10 days, effectively reduced acute and chronic pain in patients who underwent this intervention. Furthermore, it was observed that the pain level of patients in the control group, who did not receive any intervention, significantly increased during 10 days, which may be caused by the disease progression. Deep diaphragmatic breathing could improve acute and chronic pain control among GI cancer patients in the presence of unknown definitive factors. Our study results can serve as a foundation for related research and nursing care plans for these patients. Additionally, in future studies, it is advisable to explore the application of this technique as a non-pharmacological approach to address other common problems experienced by cancer patients, such as sleep disorders and fatigue, additionally, we suggest conducting further studies to explore the impact of deep diaphragmatic breathing on various complications experienced by cancer patients, such as quality of life, respiratory discomfort, lack of energy, and gastrointestinal issues.

## CRediT authorship contribution statement

**Maryam Rezaei:** Writing – review & editing, Writing – original draft, Data curation, Conceptualization. **Nader Salari:** Writing – review & editing, Writing – original draft, Visualization, Software, Formal analysis, Conceptualization. **Mozafar Aznab:** Writing – review & editing, Writing – original draft, Data curation, Conceptualization. **Sayed Vahid Jasmi:** Writing – review & editing, Writing – original draft, Methodology, Conceptualization. **Alireza Abdi:** Writing – review & editing, Writing – original draft, Visualization, Validation, Supervision, Resources, Project administration, Methodology, Investigation, Funding acquisition, Conceptualization. **Shamarina Shohaimi:** Writing – review & editing, Writing – original draft, Methodology.

## Declarations

### Ethics approval and consent to participate

Research Ethics Committee of Kermanshah University of Medical Sciences with number IR.KUMS.REC.1400.759 has approved the study. We acquired the written informed consent of the hospital's officials and patients who participated in this study. All methods were performed following the CONSORT guidelines of 2010 and regulations Research and Technology Deputy of Kermanshah University of Medical Sciences.

## Consent for publication

Not applicable.

## Data availability statement

We uploaded the SPSS file of data as a supplementary file.

## Funding

The approval process and budgeting of data collection and analysis of the project were provided by the Research and Technology deputy of Kermanshah University of Medical Sciences with approval number 4000877.

## Declaration of competing interest

The authors declare the following financial interests/personal relationships which may be considered as potential competing interests:Alireza Abdi reports financial support and administrative support were provided by 10.13039/501100005317Kermanshah University of Medical Sciences. Alireza Abdi reports a relationship with Kermanshah University of Medical Sciences that includes: employment. Alireza Abdi has patent pending to N/A. The author declares no financial or non-financial competing interests. If there are other authors, they declare that they have no known competing financial interests or personal relationships that could have appeared to influence the work reported in this paper.
